# The Health and Health Insurance Implications of Climate Change in Sub-Saharan Africa: A Narrative Review

**DOI:** 10.3389/phrs.2024.1607212

**Published:** 2025-01-03

**Authors:** Ousmane Traoré, Jean Brice Tetka

**Affiliations:** ^1^ Department of Economics, Unité de Formation et de recherche en Sciences Economiques et de Gestion (UFR/SEG), Université Thomas Sankara (UTS), Ouagadougou, Burkina Faso; ^2^ openIMIS Global Initiative, Deutsche Gesellschaft für Internationale Zusammenarbeit (GIZ), Bonn, Germany

**Keywords:** climate change, implications, health, health insurance, review, sub-Saharan Africa

## Abstract

**Objectives:**

This paper aims to provide a narrative review of the implications of climate change on health and health insurance in sub-Saharan Africa.

**Methods:**

A comprehensive research was employed to carry out a complete narrative study on the subject. Thus, since September 2022 we searched for literature on the relationships between climate change, health, and health insurance on PubMed over an unbounded period. By updating the research method, the outputs cover the period 2009–2024.

**Results:**

Based on 19 key articles that focused on the implications of climate change for health and health insurance in sub-Saharan Africa, we highlight that climate change directly affects population health through climate-related disease. Indirectly, climate change affects health through its disruption of food availability and agriculture and through demographic shifts.

**Conclusion:**

Finally, this narrative review suggests appropriate strategies to combat the health consequences of climate change and to improve universal health insurance systems.

## Introduction

Climate change is currently one of the most pressing environmental concerns in sub-Saharan Africa (SSA), where profound climate-related stresses are expected in the coming years. Among these stresses and challenges are adverse health outcomes [1]. In anticipation of such negative health outcomes, countries in SSA are beginning to implement universal health insurance for the entire population. African countries already face special challenges in the field of healthcare provision, given the current “double burden” of decreasing effectiveness of tools against traditional tropical diseases and increased prevalence and incidence of noncommunicable diseases (NCDs) [2]. The development of universal health insurance systems in most African countries is one potential measure for addressing these health-system challenges. Concomitantly, global climate change, which affects various geographic regions at different levels, is also associated with a wide range of human health problems [1]. These pose a burden to health systems, with implications for access to care and emergency care service disruptions, especially in regions such as SSA [3]. Indeed, SSA has been identified by the Intergovernmental Panel on Climate Change (IPCC) as being the region most vulnerable to climate change impacts [4]. The African continent is particularly vulnerable to the effects of extreme weather events due to an already overburdened health system, a lack of early warning signs, poverty, inadequate infrastructure, and variable adaptive capacity [5]. Over the coming decades, SSA will face profound stresses and challenges from global climate change, many of which will manifest as adverse health outcomes [1]. Shezi et al. [6] highlight that vulnerable population groups in South Africa, especially people living in poverty, young children, women, the older population, and people with pre-existing diseases, are susceptible to new or exacerbated health threats resulting from climate change.

The intersection of health and climate change is often absent or under-represented in sub-national government strategies [7]. It is critical that impacts to health systems from climate change are well understood, especially in light of the plans to implement South Africa’s National Health Insurance (NHI) program [8].

To this end, many challenges must be addressed in relation to an exhaustive list of health drivers related to climate change. Health has many drivers that can be classified based on demographic, socio-economic, and environmental factors. Like all environmental problems of global concern, climate change remains a reality that affects societies on a broad scale [9]. Moreover, it is widely accepted and increasingly recognized within the global scientific community that climate change is a fact and that it is having an impact on human health [10]. While climate change is adversely affecting the health of people around the world, the worst affected people are those in low-income countries [11, 12]. Wright et al. [13] highlight the risks to health due to several alterations in the climate of South Africa. These include an increase in ambient temperature, which has caused a significant rise in morbidity and mortality; heavy rainfall that has led to changes in the prevalence of vector-borne diseases; drought-associated malnutrition; and exposure to dust storms and air pollution that has led to the potential exacerbation of respiratory diseases.

Through its effects on the health of the population, climate change may also affect health insurance. Attention should therefore be focused on the role played by climate change when conceptualizing or analyzing health insurance systems across the world. Weather, climate, and climate change are affecting human health, with scientific evidence increasing substantially over the past two decades, but only very limited research has been conducted on low- and middle-income countries [14].

The specific aims of this review were to 1) search, select, appraise, and synthesize published research on the health and health insurance implications of climate change in SSA; 2) highlight the key channels or categories of the consequences of climate change for health and health insurance; and 3) make recommendations for efficient health provision for countries in SSA.

## Methods

The paper provides a review of the health and health insurance implications of climate change. This review primarily sought hard evidence of the implications of climate change on health and health insurance. Analyses of a causal effect between variables or indicators of climate change and health were not considered. We conducted our academic search on PubMed, with “climate change,” “health,” and “health insurance” serving as the key search terms.

## Results

### Global Overview of the Health Impacts of Climate Change in SSA

Table 1 highlights some key examples of research focused on the implications of climate change on health and health insurance in sub-Saharan Africa. For each study, we indicate the manifestations of climate change and its potential impact pathways on health and health insurance.

**TABLE 1:** Some key research on climate change and its implications for health in sub-Saharan Africa (Ouagadougou, Burkina Faso. 2024).

Author and year	Region/country	Study design (data collection process)	Manifestations of climate change	Potential impact pathways of climate change on health and health insurance
Ramin et al. (2009) [1]	Sub-Saharan Africa	Five hypothetical cases	Discrete manifestations of climate change	Impacts on agriculture and food security, droughts, floods, malaria, and population displacement
Opoku et al. (2021) [3]	Six African countries (Ghana, Nigeria, South Africa, Namibia, Ethiopia, and Kenya)	Cross-sectional study	The study evaluated the levels of information, knowledge, and perceptions of public health professionals	Rise of vulnerabilities to climate change, focusing on its impacts on human health
Rother et al. (2020) [4]	Sub-Saharan Africa	Review	Increase in extreme weather events, such as storms, floods, droughts, heat waves, wildfires, and landslides	Mental health of children and adolescents
Theron et al. (2022) [5]	Africa	Scoping review	Climate change’s global public health emergency	Implications for access to care and emergency care service disruptions
Shezi et al. (2019) [6]	South Africa	Cross-sectional survey	Not specified (climate change–related knowledge, practices, and perceptions)	Vulnerable population groups, especially people living in poverty, young children, women, the older population, and people with pre-existing diseases
Godsmark et al. (2019) [7]	Western Cape province of South Africa	Methodological framework	Local climatic projections (warmer and potentially drier future with increased frequency and intensity of extreme weather events)	Mental health, noncommunicable diseases, injuries, poisonings (e.g., pesticides), food and nutrition insecurity–related diseases, water- and food-borne diseases, and reproductive health
Dos Santos et al. (2022) [8]	South Africa	20 key expert interviews	Not specified	Previously poor communities are most at risk for the impacts of climate change on health, as well as those with underlying medical conditions; climate change may serve as a catalyst for improving the healthcare system overall (specifically health insurance)
Rother (2020) [12]	Urban areas of sub-Saharan Africa	Framework of climate-sensitive noncommunicable diseases and achieving the Sustainable Development Goals	Environmental risk factors (e.g., pollution, chemicals) for noncommunicable diseases linked to climate change	Impact of environmental factors on noncommunicable diseases
Wright et al. (2021) [13]	South Africa	Unspecified	Increase in ambient temperature, heavy rainfall, and exposure to dust storms and air pollution	Rise in morbidity and mortality, changes in the prevalence and occurrence of vector-borne diseases, drought-associated malnutrition, and exacerbation of respiratory diseases
Nilson et al. (2021) [14]	Sub-Saharan Africa and South Asia	Narrative review	Rising temperatures, rising sea levels, and an increase in extreme weather events	Health impacts of vulnerable regions and populations
Chaix et al. (2022) [15]	Unspecified	Multidisciplinary analysis	High temperatures and extreme climate events (increase in sea levels)	Increased mortality, accidents at work, suicides, domestic violence, and assaults; development of skin cancers; modification of geographic distribution of some vector-borne diseases and aquatic bacteria (e.g., cholera outbreaks)
Smith et al. (2014) [16]	Unspecified	Review	Shifts in weather patterns and other aspects of climate change (changes in temperature and precipitation and occurrence of heat waves, floods, droughts, and fires)	Ecological disruption brought on by climate change (e.g., crop failures, shifting patterns of disease vectors), social responses to climate change (e.g., displacement of populations following prolonged drought)
Dupar (2020) [17]	Africa	Special report	Extreme weather events, including heat waves, droughts, and precipitation	The distribution of land biodiversity, the mix of plant and animal species in ecosystems, the structure and productivity of vegetation, and nutrient and water cycles
Robineau (2019) [18]	Unspecified	Report (relationships between insurance and climate change)	Sea-level rise, rising temperatures, and desertification	Difficulties in identifying risks, difficulties related to the reversal of insurers’ production cycles, and the rise of the normative power of insurers
OMS (2022) [19]	World	Report (qualitative assessment of vulnerability and adaptation)	Extreme weather events (droughts, floods, heat waves), ecosystem changes, water scarcity, changes in food availability, and changes in the distributions of vectors	Climate-sensitive diseases: cardiovascular diseases, acute and chronic respiratory diseases, acute diarrhea, mental disorders, vector-borne diseases, malnutrition, and trauma
Andersen et al. (2021) [20]	Informal settlement (urban slum) of Mukuru in Nairobi, Kenya	Three focus group discussions and five in-depth interviews conducted with a total of 28 participants	Not specified (explored knowledge and perspectives on climate change and health-related issues, with a particular focus on noncommunicable diseases)	Climate change–related diseases, nutrition and access to clean water, environmental risk factors, urban planning and public infrastructure, economic risk factors, vulnerable groups, and adaptation strategies
Sorgho et al. (2021) [21]	West Africa (Burkina Faso)	35 semi-structured, qualitative, in-depth interviews	The study identified the perceptions of climate change and health adaptation among relevant stakeholders, decision-makers, and policymakers	Water quality and quantity, heat stress, food supply and safety, vector-borne diseases, and air quality

**FIGURE 1:**
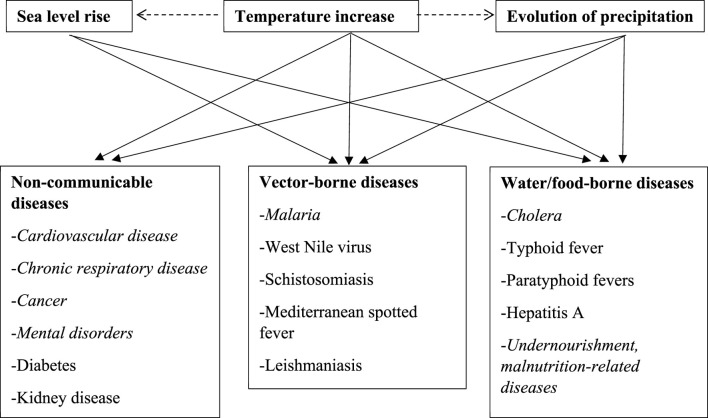
Linking population diseases to climate change (Ouagadougou, Burkina Faso, 2024). Legend: text in italics represents cases clearly identified in the included studies.

The health impacts of climate change occur mainly as a result of increasing temperatures, rising sea levels, and an increased frequency of extreme weather events. These phenomena interact with demographic, socio-economic, and environmental factors, as well as access to and the quality of healthcare, to affect the magnitudes and patterns of risks [14]. We distinguished between direct and indirect climate change–induced health impacts. Direct effects can be further classified into two types of disease: 1) NCDs and 2) vector-borne diseases. Indirect effects occur via 3) water- or food-borne diseases. Figure 1 summarizes the links between the main manifestations of climate change and the deterioration of population health. Climate change alters population health through the transmission of NDCs, vector-borne diseases, and water- or food-borne diseases. Specifically, we highlight how increased temperatures significantly contribute to the transmission of these three types of diseases. The evolution of precipitation as a possible manifestation of climate change also contributes to the spread of all three types of diseases. Moreover, sea-level rise was found to increase the transmission of vector-, water-, and food-borne diseases. The following section explains the mechanisms by which climate change causes these three main types of disease.

### Mechanisms of the Health Impacts of Climate Change in SSA

#### Relationship Between NCDs and Climate Change in SSA

Research continues to highlight the link between climate change and health outcomes. There is, however, limited evidence of the effectiveness of policies in lessening the impact of environmental factors on NCDs for people living in urban areas of SSA. Indeed, 80 percent of NCDs occur in low- and middle-income countries, and they are linked to one-third of deaths in SSA [12]. It is unclear how these statistics would change if environmental risk factors for NCDs (e.g., pollution, chemicals) linked to climate change were prevented and controlled [12]. Heat waves can affect human health through the physiological effects of heat, including diseases of the circulatory system (e.g., hypertension, atherosclerosis, ischemic heart disease, myocardial infarction, heart failure, myocarditis) and diseases of the respiratory system (e.g., pneumonia, bronchitis) [22]. Furthermore, extreme heat is associated with increased mortality, accidents at work, suicides, and domestic violence and aggression (mental disorders) [15]. The review revealed that several NCDs are related to climate change in SSA: cardiovascular disease, chronic respiratory disease, cancer, and mental disorders.

#### Vector-Borne Diseases Related to Climate Change in SSA

Climate change is altering the epidemiology of vector-borne diseases and the spread of neglected tropical diseases, such as malaria, dengue, and chikungunya [23]. The review showed the following trends occurring in SSA as a consequence of climate change: an increase in the number of cases of intestinal infections, which have a considerable seasonal manifestation, with regular records in many countries in recent years; a resurgence of diseases (e.g., malaria, yellow fever); fluctuations in daily and weekly morbidity; and an increase in the frequency of hospitalizations during hotter summers (i.e., during the rainy season in most countries in SSA) [22]. As the incidences of malaria and dengue in SSA have been very high during recent years, it is essential to improve the coverage of programs designed to increase access to safe and effective drugs and diagnostics for society’s poorest members [23].

#### Water- and Food-Borne Diseases Related to Climate Change in SSA

Human health is indirectly affected by changes in human behavior, which can increase the frequency of food-borne diseases, or other phenomena, such as floods or droughts [12]. More specifically, cholera outbreaks have been linked to variation in temperature and rainfall and in other variables, including sea and river levels [16]. In addition, climate change indirectly affects the health of a population by profoundly disrupting diet (e.g., undernourishment, malnutrition), food production systems (e.g., use of pesticides and chemical fertilizers), and the spatial distribution of the population (e.g., population migration).

Global warming is already reducing crop productivity and disrupting food systems. In recent years, yields of staple crops such as maize, wheat, and sorghum, as well as fruit crops such as mangoes, have declined across Africa, which has increased food insecurity [17]. Food insecurity and undernourishment, in turn, can jeopardize a population’s health status. Indeed [24], highlights that malnutrition remains one of the most serious socio-economic and health problems in SSA and particularly affects the poorest members of the population, as well as women and children.

The Food and Agricultural Organization (FAO) and the World Health Organization (WHO) have jointly emphasized the need for an effective risk assessment of food additives, food contaminants, natural toxicants, and residues of pesticides in agricultural products [25]. These types of risks are likely to be caused and exacerbated by climate change events. Indeed, climate change affects the agricultural sector, which must already contend with variability in rainfall in SSA. However, Chaix and Slama [15] highlight that measures to combat and adapt to climate change can also influence health. These concern urban environments and the agricultural, transportation, energy, and industrial sectors (the main emitters), which shape major health determinants (e.g., physical activity, diet, air and noise pollution, chemical contaminants). While adaptative solutions are varied, examples such as changing crops to more heat-resistant strains or increasing the use of fertilizers and herbicides could cause new pathologies, particularly carcinogens and cardiovascular diseases.

In the face of the harmful effects of climate change, forced migration also carries a very high social cost. Migration and other population movements can lead to the introduction or reintroduction of diseases, especially when displaced populations live in temporary housing with inadequate sanitation and water purification and where there are poor water storage practices and limited access to healthcare [23].

### Key Findings on the Health Insurance Implications of Climate Change

Climate change challenges the insurance industry because it involves uncertainty: risks are changing in terms of frequency, intensity, and diversity, and it is impossible to measure the extent of these changes [18]. Risks are also changing in diversity. Indeed, Gostimirovic et al. [26] highlight that, with climate change, a population can experience a decline in health status, since extreme ambient temperature affects the pharmacokinetic parameters of many cardiovascular drugs.

Therefore, climate change will challenge the insurance business by making cost containment difficult. This is because climate change alters disease risk and increases the difficulty of identifying and measuring it. Robineau [18] highlights that this change in risk configuration makes it impossible to reverse the insurance production cycle and to determine premiums in advance.

Dos Santos et al. [8] conducted the only study that questioned the impact of climate change on health insurance in SSA. The analysis focused on expert perceptions, and the results indicated that participants had no clear sense of how the organization and functioning of the NHI system of South Africa should address the health implications of climate change. There was, however, an indication that medication, mental health, and other morbidity and mortality issues would need to be factored into the NHI [8].

### Abstract of Findings

**FIGURE 2:**
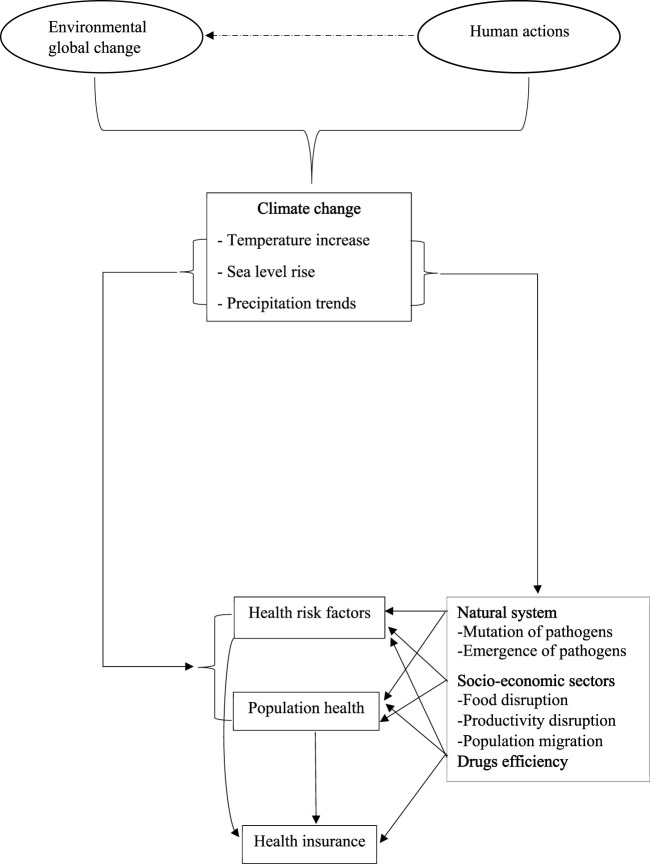
Key findings graphical abstract (Ouagadougou, Burkina Faso. 2024).

In summary, the pathways through which climate change affects health and health insurance can be grouped into three categories: the direct effects of weather events, the indirect effects of natural systems, and the indirect effects mediated by humans [16]. We provide a graphical abstract depicting the results of the literature review of the effects of climate change on health and health insurance (Figure 2). Climate change is driven by human actions and global environmental change. The main manifestations of climate change are increased temperatures, rising sea levels, and changes in precipitation. On the one hand, these manifestations can directly affect health risk factors and population health, making health insurance systems increasingly vital. Indeed, analyses of health risk factors are essential for health insurance implementation. On the other hand, they can indirectly affect health through their impact on the natural world, including by accelerating the mutation and emergence of pathogens and by affecting food availability, food technologies, and productivity technologies. Finally, climate change can adversely affect the pharmaceutical industry by limiting drug efficiency [26].

## Discussion

Globally, the discussion of health and climate change adaptation strategies by sub-national or provincial governments is often limited [7]. This literature review highlights that climate change is associated with a wide range of human health problems, which pose a burden to health systems, especially in regions such as Africa.

Through its direct and indirect effects on population health, climate change may impose challenges on health insurance systems. Across the African continent, public health systems are already under severe pressure, partly due to fragile socio-economic conditions [3]. At the same time, disasters related to climate change and gradual changes in temperature and precipitation are expected to result in an average annual loss of 3.2 percent of gross domestic product (GDP) for Africa as a whole by 2050 [27]. Additionally, Maino et al. [28] found that the effect of a 1°C rise in temperature decreases *per capita* income growth in fragile states in SSA by 1.8 percentage points. In this context, adequate funding is needed to maintain the essential functions of the healthcare system, especially in times of crisis, such as this period of climate change. Indeed, for funding sources, whether national or international, to be appropriate and effective, they must not lead to major trade-offs between sectoral budget allocations within countries, nor to a fashion or imitation effect between countries in the mobilization of resources. In the case of countries’ national adaptation plan to face climate change effect on health, Hounkpatin et al. [19] highlight that Benin prioritizes heat, drought, and flooding. But in this case, the authors find that the dominant role of global agencies in financing health system adaptation planning contributes to policy mimicry. In such a context, the various external supports could lead to a narrower focus that does not fully meet Benin’s needs in terms of climate shocks and adaptation priorities [19].

In addition to funding for essential healthcare and public health services (e.g., water, sanitation, environmental health, disaster preparedness, and health emergencies), there is a need for insurance and replacement costs for health equipment that has been destroyed or damaged by extreme weather events [29]. Improving the basic knowledge of healthcare institutions to help them better respond to a changing climate is also recommended [3].

In addition, climate change, including very high temperatures, can make the supply or use of specific drugs, vaccines, and diagnostic tests more expensive in heat-exposed regions. The storage of these products, often at low temperatures, can result in significant costs for health systems or individuals.

Thus, it appears that climate change may be an important factor in the failure of cost containment in a health insurance system, which is a prerequisite for achieving the objectives of insurance. As such, government intervention in insurance systems will be required to regulate and define more appropriate health insurance financing mechanisms that integrate the reality of climate change.

Because of the effects of climate change on health risk factors, a specific challenge for health insurance system management is to define climate change as a variable to be included in risk adjustment schemes. Indeed, the results of Dos Santos et al. [8] underscore this compelling need for health insurance systems. Health risk adjustment (or risk equalization) is a mechanism for classifying the risk profiles of insurers in order to provide subsidies or equalization levies adjusted to the risk of their insured members. It is a functional mechanism of health insurance systems that ensures equity and cost containment [30]. Thus, region- and population-specific vulnerability related to climate change must be defined as subsidy risk factors in the management of the consequences of climate change. Other seasonal variation in health expenditures must also be subsidized by risk equalization funds. Hence, the previous estimation and decomposition of annual or periodic health expenditures can be done in a way that allows for seasonal variation related to climate effects.

Climate change, through its disruption of agricultural systems, may reduce the sources of funding for these subsidies in economies where the agricultural sector substantially contributes to GDP. As such, some countries in SSA will have to find a sustainable method of resource mobilization for their health insurance systems. In such systems, government subsidies for vulnerable individuals, which are essential to the effectiveness of universal health insurance in developing countries [31], must also be strengthened in response to climate change crises. One solution is to opt for a tax on products that are harmful to the health of the population, such as tobacco, alcohol, sugar, and oils. Taxing products or activities that generate or accelerate climate change, including aviation, polluting industries, and the use of internal combustion vehicles, could provide an alternative source of funding. Finally, the real estate and land sector, which is still largely untaxed in many countries in SSA, could generate significant additional revenue, even with low tax rates. One important strategy to adapt to climate change–associated health risks is to provide training for local communities, thus ensuring adaptation strategies and climate change advocacy [20].

### Conclusion

Climate change constitutes one of the greatest threats to human health and requires political awareness for effective and efficient adaptation planning [21]. Weather, climate, and climate change are affecting human health, as has been shown by a growing body of scientific evidence over the past two decades. However, there continues to be very limited research on the implications of climate change for low- and middle-income countries [14]. More specifically, to the best of our knowledge, only one previous paper attempted to link climate change and health insurance in SSA. Therefore, our review serves as a meaningful contribution to the exploration of the possible mechanisms of the effects of climate change on health insurance and highlights the key challenges faced by health insurance systems in SSA in the context of a changing climate. Three main challenges were underlined for countries in SSA. First, they must ensure the successful operation of the core functions of healthcare systems in the face of climate change. Second, they must define the climate change–related risk factors to be included in health insurance risk adjustment schemes. Third, they must acquire adequate funding for their health insurance systems. Most countries in sub-Saharan Africa are in the process of experimenting with or implementing health insurance systems. Thus, the embryonic nature of health insurance systems in the region is a limitation of our research, insofar as there is very little literature to draw major lessons on the adaptation of these health insurance systems to the effects of climate change.

## Abbreviations

FAO, Food and Agricultural Organization; GDP, Gross domestic product; IPCC, Intergovernmental Panel on Climate Change; NCDs, Non-communicable diseases; NHI, National Health Insurance; PRISMA, Preferred Reporting Items for Reviews and Meta-Analyses; SSA, Sub-Saharan Africa; WHO, World Health Organization.
